# Notes and News

**Published:** 1896-09-26

**Authors:** 


### NOTES AND NEWS.

(Collected and Communicated.)

The Metropolitan Asylums Boards are contemplating the purchase of a site for their new offices upon the Victoria Embankment. The price asked is £54,000, which the Local Goverment Board regard as too expensive, and their consent consequently has not yet been obtained.

We have been informed by the secretary of the Derbyshire Royal Infirmary that the committee of that institution have now adopted the uniform system of accounts. We congratulate the hospital upon this last step of its progress, showing as it does that the Derbyshire Royal Infirmary means to occupy the place to which it is entitled in the first rank of the hospitals of our country, and as a model of efficiency and good management. We are glad to learn also that the Evans wing is nearly ready for occupation, thus completing the additions for the present.

The new Leith Public Health Hospital; in other words, the fever hospital, is now formally opened. A site of nine acres has been obtained, which allows of recreation grounds for patients and for nurses. The buildings, which are plain in appearance, are of brick. They consist of administration block, an isolation block, and four ward pavilions, placed due south and north. The administration block consists of three storeys. The isolation block contains two wards with nursing accommodation and offices, and is intended for ten patients under observation. The pavilions each contain two wards for ten patients in each, and offices. Two thousand cubic air space is allowed per patient. The gate-keepers' lodge provides waiting and dressing rooms for visitors, where garments to be worn when visiting patients are provided. Besides bath-rooms, moveable baths have been provided. The cost of the hospital is estimated at £50,000.

The new Bridgenorth and Shropshire Infirmary was opened last week by the Viscountess Boyne. The buildings cost £6,099.

An outbreak of typhoid fever is reported at Leeds. It is attributed to the milk supply in one part of the town. A temporary iron hospital is to be erected.

Dr. Samuel Fenwick has been elected consulting physician to the London Hospital on his retirement from the post of physician, which he has held for seventeen years.

Lord Justice Lindley will distribute the prizes to the students of St. Thomas's Hospital Medical School on Friday, October 2nd, at three o'clock, at the hospital.

The ninth Congress of Italian Alienists will be held on October 5th at Florence. The annual French Congress of Alienists and Neurologists will be held at Toulouse next year.

The sum of £11,930 has now been received by the Metropolitan Hospital Saturday Fund as the result of this year's collection. The workshops and business houses contributed £7,150, and the ladies' street collection £4,780.

The Biennial Festival Dinner of Guy's Hospital will this year be held at the Hôtel Cecil, where past and present students and their friends will meet under the chairmanship of Dr. Frederick Taylor on Thursday, October 1st.

Professor Michael Foster will deliver the first "Huxley Lecture" on recent advances in science, and their bearing on medicine and surgery, at Charing Cross Hospital, on Monday, October 5th, 1896, at four o'clock.

The County Council of the West Riding of Yorkshire are setting a good example in providing every place with a suitable room in which to hold inquests, instead of permitting the use of public-houses, which is the general custom.

A conversazione will be held at St. Mary's Hospital on Friday, October 2nd, in aid of the new out-patients' department. An excellent programme of music and other entertainments has been arranged, and tickets (2s. 6d. each) can be procured from the dean at the hospital.

Many struggling members of the medical profession will see the advantage of the action taken by some medical practitioners in Kentucky. They have followed the example of the tradesmen of the district, and demand cash payment for their services; that is to say, payment within thirty days. The public frequently show an astounding want of conscience where the doctor is concerned. The well-to-do have been heard to declare that they cannot afford to pay their doctor, whilst others will pay a small sum on account, if a claim is urged. Custom, at the same time, renders it very difficult for a medical man to relinquish attendance upon a patient only because he does not pay, and it is equally difficult for a doctor to sue a delinquent patient.

